# A Chitosan Coating Containing Essential Oil from *Origanum vulgare* L. to Control Postharvest Mold Infections and Keep the Quality of Cherry Tomato Fruit

**DOI:** 10.3389/fmicb.2016.01724

**Published:** 2016-11-08

**Authors:** Tainá A. Barreto, Sonalle C. A. Andrade, Janeeyre F. Maciel, Narciza M. O. Arcanjo, Marta S. Madruga, Bruno Meireles, Ângela M. T. Cordeiro, Evandro L. Souza, Marciane Magnani

**Affiliations:** ^1^Laboratório de Processos Microbianos em Alimentos, Departamento de Engenharia de Alimentos, Centro de Tecnologia, Universidade Federal da ParaîbaJoão Pessoa, Brasil; ^2^Laboratório de Flavor, Departamento de Engenharia de Alimentos, Centro de Tecnologia, Universidade Federal da ParaíbaJoão Pessoa, Brasil; ^3^Laboratório de Combustíveis, Universidade Federal da ParaíbaJoão Pessoa, Brasil; ^4^Laboratório de Microbiologia de Alimentos, Departamento de Nutrição, Centro de Ciências da Saúde, Universidade Federal da ParaíbaJoão Pessoa, Brasil

**Keywords:** *Lycopersicon esculentum* L., edible coatings, phenolic compounds, storage quality

## Abstract

The efficacy of an edible chitosan coating (CHI; 4 mg/mL) and *Origanum vulgare* L. essential oil (OVEO; 1.25 μL/mL) for maintaining the quality of cherry tomato fruit during storage at room (25°C; 12 days) and cold (12°C; 24 days) temperatures was assessed. CHI and OVEO in combination showed *in vitro* fungicidal effects against *R. stolonifer* and *Aspergillus niger*. CHI-OVEO coating reduced the incidence of black mold and soft rot caused by these fungi in artificially contaminated cherry tomato fruit during storage at both temperatures. CHI-OVEO coating delayed the appearance of the first visible signs of black mold and soft rot in artificially contaminated cherry tomato fruit stored at room temperature by 6 days and by more than 9 days in those stored at cold temperature. At the end of storage at room and cold temperature fruit coated with CHI-OVEO showed higher firmness (>2 N/mm) and lower weight loss (>2%) compared to uncoated tomato fruit. CHI-OVEO coating delayed the decrease of lycopene, ascorbic citric acid, glucose and fructose during the storage time assessed at room or cold temperatures. The increase of catechin, myricetin, caffeic and syringic acids was higher (1–9 mg/g) in cherry tomato fruit coated with CHI-OVEO compared to uncoated fruit during the storage at both temperatures studied. CHI-OVEO coating is a feasible treatment for maintaining the storage quality of cherry tomato fruit.

## Introduction

Cherry tomato (*Lycopersicon esculentum* L.) fruits have increased in popularity due to their high content of sugars and health promoting compounds as well as their convenience of use; they are consumed either as an ingredient (such as in salads) or alone (D'Aquino et al., 2016; Wu et al., 2016).

The infection of cherry tomato fruit with the pathogenic fungi *Aspergillus niger* and *Rhizopus stolonifer* is associated with most postharvest losses and decreased storage quality in these fruit (Fagundes et al., 2015a). Black mold, caused by *A. niger*, is a postharvest disease characterized by softening and darkening of the fruit infected site, followed by breaking of the peel and development of a dark mycelia (Plascencia-Jatomea et al., 2014). *R. stolonifer* is the etiological agent of the soft rot in cherry tomato fruit, a disease recognized by watery areas covered by coarse, gray hairy mycelium that forms a mass of black sporangia at its tips (Bautista-Baños et al., 2008).

Postharvest decay caused by fungal contamination of cherry tomato fruit has been primarily controlled by the application of synthetic fungicidal agents in the field and during the postharvest period (Fagundes et al., 2015b; Guerra et al., 2015). These agents do not adversely affect the appearance or quality of the treated fruit (de Amiri et al., 2008), but their indiscriminate and excessive use on crops has been a major cause of the development of resistant fungal pathogen populations (de Oliveira et al., 2014). In addition, there is an increased awareness of the potentially harmful nature of these chemical compounds on human health and the (Aquino et al., 2015; FAO, 2016).

Edible coatings based on polysaccharides (e.g., hydroxypropylmethylcellulose, locust bean gum, cassava starch or chitosan) are considered environmentally friendly alternatives and can reduce the use of chemical preservatives applied for postharvest treatments (El-Anany et al., 2009; Ali et al., 2011). Chitosan (CHI) has favorable characteristics for use as an edible coating because it is a biodegradable cationic hydrocolloid that possess antifungal activity in addition to its film-forming ability (Elsabee and Abdou, 2013; Shao et al., 2015).

The incorporation of essential oils (EOs) in CHI-coatings reduces the water permeability and can improve the antimicrobial efficacy because the EO constituents are released onto the fruit surface over time (Sánchez-González et al., 2011). The EOs from *Origanum vulgare* L. (oregano—OVEO) possess strong inhibitory effects against a variety of phytopathogenic fungi and do not seem to cause the development of resistance in microorganisms (Luz et al., 2012; Santos et al., 2012; Llana-Ruiz-Cabello et al., 2015).

A previous study showed interesting antifungal effects when OVEO was incorporated into CHI-coatings to control mold decay in grapes (Santos et al., 2012). However, the efficacy of CHI-coating containing OVEO for controlling black mold and soft rot and for preserving the storage quality of cherry tomato fruit is unknown. Thus, the present study was performed to assess (i) the *in vitro* effects of CHI and/or OVEO on mycelial growth, spore germination and sporulation of *A. niger* and *R. stolonifer*; (ii) the efficacy of a coating comprised of CHI and OVEO to control dark mold and soft rot in cherry tomato fruit during storage at room and cold temperature, and (iii) the effects of this coating on cherry tomato fruit quality parameters.

## Materials and methods

### Materials

Chitosan (CHI) of medium molecular weight (deacetylation degree 75–85%, batch 03318AJ) was obtained from Sigma-Aldrich Co. (St. Louis, USA). *Origanum vulgare* L. essential oil (OVEO) (Batch ORE001 density at 20°C: 0.90, refractive index at 20°C: 1.47) was supplied by Aromalândia Ind. Com. Ltda. (Minas Gerais, Brazil). The GC–MS analysis of the OVEO was performed following previously described procedures (Targino et al., 2016). A total of 22 different constituents were identified in the OVEO. The constituents present in the highest amounts in OVEO were carvacrol (64.42%), σ-cymol (12.50%), and γ-terpinene (6.78%). Other constituents, such as thymol (3.82%), linalol (2.85%), and α-pinene (1.69%) were found in minor amounts (Supplementary Table 1).

Mature, commercially available cherry tomato (*Lycopersicon esculentum* L.) fruit were purchased from EMPASA (Supplies and Services Company of Paraiba, João Pessoa, Brazil). Fruit of similar shape and color and without signs of fungal infection or mechanical damage were selected for the experiments. Prior to the tests, the cherry tomato fruit were disinfected via immersion in a sodium hypochlorite solution (1 mL/100 mL; pH 7.2) for 5 min, rinsed with sterile water and dried for 2.5 h in a Class II safety cabinet (de Sousa et al., 2013).

### Fungal strains

*R. stolonifer* URM 3728 and *A. niger* URM5162 were obtained from the Mycology Culture Collection of the Federal University of Pernambuco, Recife, Brazil. Stock cultures were grown in Sabouraud agar (Himedia, India) at 25°C for 7 days to allow for sufficient sporulation. Spores were collected in a sterile saline solution (0.85 g/100 mL NaCl), and the suspension was filtered through triple gauze to remove hyphal fragments. The number of spores was determined with a hemocytometer and the spore count was adjusted with a sterile saline to yield an inoculum of approximately 10^6^ spores/mL (Guerra et al., 2015).

Prior to the assays, each test fungal strain was tested for its capability to cause dark mold or soft rot infection in cherry tomato fruit by looking for a characteristic set of symptoms. After black mold or soft rot development, the strains were re-isolated and identified with taxonomical keys. Continuous re-inoculations and re-isolations on cherry tomato fruit were carried out to maintain the pathogenicity of the test fungal strains (Plascencia-Jatomea et al., 2014; Guerra et al., 2015).

### Preparation of CHI and/or OVEO solutions

CHI solutions were obtained by dissolving the powder (16 mg/mL) in acetic acid (1 mL/100 mL) with stirring (120 rpm) at room temperature (28°C) for 24 h (Sánchez-González et al., 2011). Successive serial dilutions (1:1) were performed in Sabouraud broth (Himedia, India) to obtain solutions of different concentrations (16, 8, 4, 2, and 1 mg/mL). The pH of the CHI solutions used in the *in vitro* antifungal activity assays was adjusted to 5.0 by adding 1 M NaOH. OVEO solutions were obtained by dissolving the substance (80 μL/mL) in Sabouraud broth containing Tween 80 [2%, v/v (Himedia, India)] as a emulsifying agent (Santos et al., 2012), and successive dilutions (1:1) in the same broth were prepared to obtain solutions of different concentrations (20, 10, 5, 2.5, 1.25, and 0.06 μL/mL). Tween 80 at the highest final concentration tested (2%, v/v) did not interfere with the fungal growth. For the combined application, CHI was diluted in acetic acid (1 mL/100 mL) (as described above) and OVEO was added at the desired concentration. The combined solution was obtained by stirring for 18 h at room temperature (Ojagh et al., 2010).

### Determination of the minimum inhibitory concentration (MIC) of CHI and OVEO

The MIC values of CHI and OVEO against *A. niger* and *R. stolonifer* were determined by macrodilution in broth. An aliquot of 1 mL of the spore suspension (approximately 10^6^ spores/mL) was inoculated in 4 mL of double-concentrated Sabouraud broth, and 5 mL of the CHI solutions at different concentrations (16–1 mg/mL) or OVEO (20–0.03 μL/mL) were added. The mixture was incubated at 25°C for 7 days. At the end of this period, the lowest CHI or OVEO concentration that caused inhibition of visible fungal growth was considered as the MIC (Santos et al., 2012).

### *In vitro* antifungal effects of CHI and OVEO

*In vitro* antifungal effects of CHI and OVEO were measured by observing the inhibition of radial mycelial growth, spore germination and sporulation of the test fungal strains. The inhibition of the radial mycelial growth of *A. niger* and *R. stolonifer* was determined using the poisoned solid substrate technique. For this method, 2-mm disks taken from a 7-days fungal culture (grown on Sabouraud agar at 28°C) were placed on the center of Petri dishes containing Sabouraud supplemented with only CHI (4 mg/mL) or OVEO (1.25 μL/mL) or CHI (4 mg/mL) combined with OVEO (5, 2.5, and 1.25 μL/mL). The material was incubated at 28°C for 7 days and the radial mycelial growth (mm) was measured using calipers every 24 h over a 72 h period. Cultures cultivated in growth media without addition of CHI or OVEO were similarly tested (controls). The results were expressed as inhibition rates (percentage) of mycelial radial growth (mm) relative to the control (de Oliveira et al., 2014).

To assess the effects on spore germination, 0.1 mL aliquots of the *A. niger* or *R. stolonifer* spore suspensions (10^6^ spores/mL) obtained from a 7-day-old culture cultivated on Sabouraud agar at 28°C were placed in tubes with 0.1 mL of the solution containing CHI (4 mg/mL) or OVEO (1.25 μL/mL) or CHI (4 mg/mL) combined with OVEO (5, 2.5, and 1.25 μL/mL). The mixture (0.1 mL) was placed on a glass slide and statically incubated in a moist chamber for 24 h at 28°C. The slides were treated with lactophenol cotton blue stain and observed under the light microscope. On each slide, 200 spores were counted. As controls, spore suspensions obtained from fungal cultures grown in media without the addition of CHI and/or OVEO were similarly assayed. The results were expressed as the percent inhibition of spore germination by comparing the number of germinated spores in the media supplemented with CHI and/or OVEO with those obtained in the control assay (Guerra et al., 2015).

Sporulation by *A. niger* and *R. stolonifer* was evaluated according to a previously described procedure (de Sousa et al., 2013). The fungi were grown for 7 days at 25°C on Sabouraud agar or under the same conditions in Sabouraud agar supplemented with CHI (4 mg/mL) or OVEO (1.25 μL/mL) or CHI (4 mg/mL) combined with OVEO (5, 2.5, and 1.25 μL/mL). From each Petri dish containing the mycelium, three 8 mm-mycelium plugs were taken from the central and peripheral regions using a copper awl. The plugs were transferred to individual test tubes containing 10 mL of a mixture (1:1) of saline (NaCl 0.89% w/v) and Tween 80 solution (0.1% v/v). After vigorous shaking of the mixtures, the spores were counted in a Neubauer chamber. As a control, mycelium plugs not exposed to CHI and/or OVEO were similarly assayed. The results were expressed as the number of total spores relative to the control treatment.

### Application of CHI and OVEO coatings to cherry tomato fruit

The fruit (*n* = 80) was first immersed in an inoculum solution (10^6^ spores/mL) of *A. niger* or *R. stolonifer* for 1 min and then placed in a Class II biosafety cabinet for 1 h (25°C). Then, the fruit were immersed in 500 mL of a solutions composed of only CHI (4 mg/mL) or OVEO (1.25 μg/mL) or CHI (4 mg/mL) combined with OVEO (1.25 μg/mL), with mild shaking for 1 min. The fruit were air-dried and stored in a polyethylene container. One fruit group was stored at 25°C (room temperature), and the other at 12°C—the temperature commonly applied during transportation and market storage of cherry tomato fruit (Li et al., 2016). In the control experiment, CHI and OVEO solutions were replaced with sterile distilled water. Each treatment included 40 fruit. Fruits stored at room temperature were sampled at 1, 4, 8, and 12 days; those stored at cold temperature were sampled at 1, 6, 12, 18, and 24 days. The fruit were examined for characteristic signs of Rhizopus soft rot and Aspergillus black mold (Feng and Zeng, 2007; Liu et al., 2007). The results are expressed as the storage time needed for visible signs of fungal infection to appear and the disease incidence (percent of infected fruit) at the assessed time intervals (Guerra et al., 2015).

### Physicochemical analyses of cherry tomato fruit

The uncoated or coated cherry tomato fruit were evaluated for weight loss, firmness, total soluble solids (TSS), titratable acidity (TA), lycopene content and color at the same time intervals evaluated for fungal infection. To determine the weight loss of the cherry tomato fruit during storage, the fruit weight loss during each assessed period was calculated as a percentage of the initial weight (de Oliveira et al., 2014). The firmness was measured in a TA-XT2 Texture Analyzer (Stable Micro Systems, United Kingdom) using a 3 mm diameter probe (1/8). The results were expressed as N/mm (Guerra et al., 2015). Prior to the TSS, TA and lycopene analysis, 10 fruit from each group were randomly chosen, macerated and homogenized to remove the required amount of sample for each experiment. The TSS was determined using a digital refractometer (HI96801, Hanna Instruments, São Paulo, Brazil), and the results were expressed as °Brix (Ali et al., 2010). The TA was determined via titration with 0.1 N NaOH to the phenolphthalein-end point, and the results are expressed as a percent of anhydrous citric acid (Aquino et al., 2015). The peel color was measured at three different equatorial points of the fruit using the CIELab system (L^*^ a^*^ b^*^). The chroma (C^*^ab) and hue angle (h^*^ab) were measured in a Minolta colorimeter (CR-300 Co., Osaka, Japan) using a 10-mm quartz cuvette according to the International Commission on Illumination (CIE, 1986). A D65 illuminant (standard daylight) at a 10° angle was used for the CIELab color scale (L^*^a^*^b^*^). Lycopene content in the cherry tomato fruit was determined according to procedures described by Li et al. (2016). Lycopene was extracted using a mixture of hexane:acetone:ethanol (2:1:1). The lycopene content of cherry tomato fruit was measured spectrophotometrically at 472 nm using an extinction coefficient (E%) of 3450. The results were expressed in mg/100 g of fruit.

### Determination of sugars and organic acids in cherry tomato fruit

Sugars and organic acids in the cherry tomato fruit were determined at the 1st and 12th day of storage at room temperature or at the 1st, 12th, and 24th days of storage at cold temperature. From the fruit-macerate obtained for the physicochemical analyses, an aliquot of 2 g was homogenized in ultra-pure water (10 mL) for 10 min using a mini-Turrax apparatus. The mixture was centrifuged (4000 × g, 15 min, 4°C), and the supernatant was collected and filtered through a 0.45 mm membrane. Sugars (xylose, glucose, fructose and sucrose) and organic acids (ascorbic, acetic, citric, formic, lactic, malic, propionic and succinic acids) were measured in the extract by HPLC using a Varian Waters chromatograph (model 2690, Varian, California, USA) equipped with a binary solvent system “valve Rheodyne” with a 20-μL loop at a temperature of 65°C, coupled with a diode array detector (Varian 330) at wavelengths of 220–275 nm, a pumping system with high pressure gradient setting (VARIAN 230) and GALAXIE Chromatography Data System processing software. The other analytic conditions were as follows: Agilent Hi-Plex H column (7.7 × 300 mm, 8 μm); mobile phase 0.009 H_2_SO_4_; and flow rate, 0.7 mL/min. The HPLC sample peaks were identified by comparing their retention times with those of organic acid and sugar standards (Sigma Aldrich®). The results were expressed in mg per 100 g of fresh weight (Zeppa et al., 2001).

### Determination of phenolic compounds in cherry tomato fruit

Phenolic compounds in the cherry tomato fruit were determined at the same storage time intervals as the sugar and organic acid analyses. An aliquot of 5 g was homogenized in methanol (50 mL) for 4 h, stirred at room temperature on a shaker table, filtered through Whatman no. 4 paper, and dried by evaporation. The phenolic extracts obtained in this way were then dissolved in water. Phenolic acids and flavonoids were measured using a Shimadzu Prominence LG2OAT HPLC equipped with a photodiode array detector (SPDM2O) and a reversed-phase column (Shimpack CLCODS, 4.6 mm × 250 mm × 5 mm). For the benzoic and cinnamic acid derivatives (2,4-dihydroxybenzoic, 3,4-dihydroxybenzoic, ferulic, ellagic, *trans*-cinnamic, *p*-coumaric, syringic), the mobile phase consisted of a mixture of 2% aqueous acetic acid in water (A) and acetonitrile: methanol (2:1) (B) at a flow rate of 1 mL/min. A gradient elution was used, starting with 20% B up to 15 min, 30% B at 20 min, 40% B at 30 min and isocratic at 40% B up to 45 min. The flavonoids (myricetin, quercetin, catechin, rutin, kaempferol, hesperetin, naringenin, chrysin) were separated using a mobile phase consisting of 1% aqueous acetic acid (A) and methanol (B) at a flow rate of 1 mL/min. The mobile phase was delivered using the following solvent gradient: 0–3 min 40% B; 5–15 min 45% B; 17–25 min 50% B; 27–35 min 55% B; and 35–40 min 70% B. The injection volume was 10 μL. The identification and quantification of phenolic compounds was based on the retention times, the UV spectra and a chromatographic comparison (co-injection) with authentic markers from Sigma Aldrich®. The results were expressed as changes in mg per g of fresh weight during the storage time (Borges et al., 2011).

### Sensory analysis

Uncoated or coated cherry tomato fruit were subjected to sensory tests at the 1st, 6th, and 12th days of the cold temperature storage (to ensure the microbiological safety of the samples offered to the panelists). The acceptance and preference tests were performed with 100 untrained panelists after approval from an Ethics Research Committee (protocol 712.884/2014). The tests were performed in individual booths under controlled temperature and lighting conditions. Each panelist received the uncoated or coated cherry tomato fruit served on white plates codified with a three-digit number in a blinded and random sequence. The panelists were advised to eat a salty biscuit and drink water between samples to avoid aftertaste interference. For the acceptance of attributes (appearance, flavor, texture, color, aftertaste and overall evaluation), a 9-point structured hedonic scale was used, ranging from one “dislike very much” to nine “like very much.” For the preference test, the panelists were oriented to select the most and least appreciated samples considering the overall evaluation. The purchase intent was assessed using a 5-point structured hedonic scale ranging from one “certainly would not buy” to five “certainly would buy” (de Sousa et al., 2013).

### Statistical analysis

All analyses were performed in triplicate on three different occasions. The data were analyzed using Sigma Stat software 2.03 (Jandel Scientific Software, San Jose, California). The results were evaluated by ANOVA followed by Tukey's *post-hoc* test or Student's *t*-test. The results were presented as the means ± standard deviation, and a probability *p* ≤ 0.05 was considered significant.

## Results

### *In vitro* antifungal effects of CHI and OVEO

CHI and OVEO exhibited MICs of 8 mg/mL and 10 μL/mL, respectively, against both *R. stolonifer* and *A. niger*. CHI was tested at 4 mg/mL (1/2 MIC) in inhibition assays of mycelial growth, spore germination and sporulation because this is the lowest concentration needed to form a viscous solution with coating features for application in cherry tomato fruit (Guerra et al., 2015).

The incorporation of CHI at 4 mg/mL combined with OVEO at 5, 2.5, or 1.25 μL/mL, or OVEO at 10 μL/mL in growth media resulted in a fungicidal effect toward *R. stolonifer* and A. *niger* with the complete inhibition of fungal mycelial growth throughout the 48 h-incubation time. During the same period of incubation, the incorporation of only CHI at 4 mg/mL or OVEO at 1.25 μL/mL in growth media inhibited less than 50% of the mycelial growth of both *R. stolonifer* and *A. niger*.

The application of CHI at 4 mg/mL combined with OVEO at 5, 2.5, or 1.25 μL/mL strongly inhibited the spore germination of *R. stolonifer* (75–84%) and *A. niger* (90–100%). Lower inhibition (*p* ≤ 0.05) of the spore germination of *R. stolonifer* or *A. niger* was observed when assays were performed with only CHI at 4 mg/mL (≤50%) or OVEO at 1.25 μL/mL (≤30%) (Supplementary Table 2).

The sporulation of the tested fungal strains was completely inhibited by CHI at 4 mg/mL combined with OVEO at 5, 2.5, or 1.25 μL/mL. Similar to the results observed in the assays of mycelial growth inhibition, there were no differences (*p* > 0.05) in the inhibition of the spore germination or the sporulation of both fungi induced by the combination of CHI with OVEO at the different tested concentrations (Supplementary Table 2).

### Effects of CHI and OVEO coatings on cherry tomato fruit

Considering that CHI combined with the different concentrations of OVEO showed similar *in vitro* antifungal effects against both *A. niger* and *R. stolonifer*, the assays with cherry tomato fruit were performed with a CHI-coating containing OVEO at 1.25 μL/mL, the lowest OVEO concentration assayed.

Cherry tomato fruit artificially contaminated and coated with CHI-OVEO presented visible signs of soft rot after 8 days of storage at room temperature, and after 21 days of storage at cold temperature (Table 1). The first visible signs of black mold on fruit coated with CHI-OVEO were observed after 8 and 15 days of storage at room and cold storage temperature, respectively. Uncoated cherry tomato fruit or coated fruit with only CHI or OVEO presented the first visible signs of black mold and soft rot in a shorter period of storage at room or cold temperatures (Table 1).

**Table 1:** **Occurrence of Rhizopus soft rot and Aspergillus black mold in cherry tomato fruit uncoated and coated with chitosan (CHI) and/or *O. vulgare* L. essential oil (OVEO) followed by storage at room temperature (25°C, 12 days) or low temperature (12°C, 24 days)**.

**Treatments**	**Days of storage for detection of first signs of mold infection**		**Percent of infected fruit at the end of the storage time**	
	**Room temperature**	**Cold temperature**	**Room temperature**	**Cold temperature**
**RHIZOPUS SOFT ROT**				
Control	2nd	9th	100.0% (±0.00)^a^	87.50% (±0.00)^a^
CHI 4	4th	15th	53.75% (±0.02)^c^	68.75% (±0.09)^b^
OVEO 1.25	4th	12th	63.81% (±0.09)^b^	67.52% (±0.00)^b^
CHI 4 + OVEO 1.25	8th	21st	12.02% (±0.00)^d^	5.05% (±0.04)^c^
**BLACK MOLD**				
Control	2nd	6th	100.0% (±0.00)^a^	87.53% (±0.00)^a^
CHI 4	4th	9th	62.75% (±0.04)^c^	52.50% (±0.04)^c^
OVEO 1.25	4th	6th	75.01% (±0.00)^b^	62.51% (±0.00)^b^
CHI 4 + OVEO 1.25	8th	15th	19.05% (±0.03)^d^	10.00% (±0.05)^d^

Control: 0 μL/mL of chitosan and essential oil; CHI 4 + OVEO 1.25: CHI 4 mg/mL + OVEO 1.25 μL/mL; a–d: For each trial, different superscript letters in the same column denote differences (p ≤ 0.05) among the mean values (for each fungus submitted for the different treatments at the same storage condition).

The incidence of black mold on cherry tomato fruit coated with CHI-OVEO at the end of the storage at room and cold temperature was 19 and 10%, respectively; whereas for the uncoated fruit the incidence was greater than 85% (Table 1). At the end of the storage time at room and cold temperatures, the incidence of soft rot on cherry tomato fruit coated with CHI-OVEO was 12 and 5%, respectively. In uncoated cherry tomato fruit, the incidence of soft rot was greater than 87% in fruit stored at room or cold temperature (Table 1). The incidence of black mold or soft rot on fruit coated with only CHI or OVEO was ≥50% for storage at either room or cold temperature (Table 1).

### Effects on the physicochemical parameters of cherry tomato fruit

The physicochemical parameters of uncoated and coated cherry tomato fruit were evaluated during storage at room (25°C) and cold temperatures (12°C). The weight loss of the cherry tomato fruit coated with CHI-OVEO was lower (*p* ≤ 0.05) than that observed for uncoated fruit or fruit coated with only CHI or OVEO (Table 2).

**Table 2:** **Mean values of the physicochemical quality parameters in cherry tomato fruit uncoated and coated with chitosan (CHI) and/or *O. vulgare* L. essential oil (OVEO), followed by storage at room temperature (25°C, 12 days) or cold temperature (12°C, 24 days)**.

**Treatments**	**Days of storage at room temperature (25°C)**				**Days of storage at cold temperature (12°C)**				
	**1**	**4**	**8**	**12**	**1**	**6**	**12**	**18**	24
**WEIGTH LOSS (%)**									
Control	0.00 (±0.00)^D^	1.99 (±0.01)^aC^	6.22 (±0.01)^aB^	10.85 (±0.02)^aA^	0.00 (±0.00)^E^	0.52 (±0.06)^aD^	2.39 (±0.06)^aC^	4.49 (±0.08)^aB^	6.21 (±0.03)^aA^
CHI 4	0.00 (±0.00)^D^	1.75 (±0.02)^bC^	6.01 (±0.04)^bB^	8.01 (±0.03)^cA^	0.00 (±0.00)^E^	0.36 (±0.06)^bD^	1.32 (±0.04)^bC^	2.23 (±0.07)^bB^	4.19 (±0.02)^bA^
OVEO 1.25	0.00 (±0.00)^D^	1.94 (±0.01)^aC^	6.11 (±0.02)^abB^	9.82 (±0.02)^bA^	0.00 (±0.00)^E^	0.49 (±0.04)^aD^	1.54 (±0.05)^bC^	2.44 (±0.03)^bB^	4.81 (±0.01)^bA^
CHI4 + OVEO 1.25	0.00 (±0.00)^D^	1.20 (±0.01)^cC^	4.37 (±0.03)^cB^	6.58 (±0.01)^dA^	0.00 (±0.00)^E^	0.30 (±0.03)^bD^	1.02 (±0.05)^cC^	1.74 (±0.02)^cB^	3.80 (±0.02)^cA^
**FIRMNESS (N/mm)**									
Control	10.55 (±0.05)^bA^	8.63 (±0.03)^bB^	7.94 (±0.07)^bC^	6.40 (±0.04)^cD^	10.15 (±0.07)^bcA^	10.01 (±0.04)^aA^	9.23 (±0.04)^bB^	8.51 (±0.03)^bC^	6.31 (±0.03)^cD^
CHI 4	8.94 (±0.05)^dA^	7.54 (±0.03)^cB^	7.47 (±0.01)^cB^	7.19 (±0.02)^bC^	10.09 (±0.04)^cA^	8.98 (±0.02)^bB^	8.61 (±0.03)^cC^	8.06 (±0.04)^cD^	7.54 (±0.03)^bD^
OVEO 1.25	9.65 (±0.02)^cA^	8.44 (±0.08)^bB^	7.30 (±0.04)^cB^	6.54 (±0.03)^cB^	10.33 (±0.03)^bA^	8.75 (±0.04)^bB^	8.34 (±0.03)^cB^	7.34 (±0.04)^dC^	6.40 (±0.04)^cD^
CHI4 + OVEO 1.25	11.36 (±0.03)^aA^	9.26 (±0.02)^aB^	8.17 (±0.05)^aC^	8.07 (±0.02)^aC^	12.68 (±0.04)^aA^	10.22 (±0.05)^aB^	9.94 (±0.02)^aC^	9.15 (±0.05)^aD^	8.77 (±0.01)^aE^
**TITRABLE ACIDITY (mg/100 g OF CITRIC ACID)**									
Control	12.39 (±0.05)^aA^	10.26 (±0.06)^aB^	9.40 (±0.03)^aBC^	8.43 (±0.03)^cC^	11.71 (±0.08)^aA^	10.59 (±0.07)^bB^	9.05 (±0.21)^bC^	8.51 (±0.05)^bD^	8.11 (±0.03)^cE^
CHI 4	9.87 (±0.09)^cA^	9.56 (±0.15)^aA^	9.50 (±0.06)^aA^	9.33 (±0.04)^bA^	11.10 (±0.01)^BA^	11.04 (±0.07)^aA^	11.02 (±0.09)^aA^	10.99 (±0.01)^aA^	10.97 (±0.01)^aA^
OVEO 1.25	9.67 (±0.01)^cA^	9.47 (±0.06)^aA^	9.37 (±0.02)^aA^	9.11 (±0.09)^bcA^	11.09 (±0.05)^bA^	10.99 (±0.01)^aB^	10.95 (±0.05)^aB^	10.93 (±0.02)^aB^	10.92 (±0.03)^bB^
CHI 4 + OVEO 1.25	10.86 (±0.14)^bA^	10.44 (±0.18)^aA^	10.37 (±0.06)^aA^	10.34 (±0.05)^aA^	11.06(±0.04)^bA^	11.09 (±0.01)^aB^	10.98 (±0.01)^aB^	10.97 (±0.02)^aB^	10.95 (±0.01)^abB^
**TOTAL SOLUBLE SOLIDS (**°**BRIX)**									
Control	7.15 (±0.07)^aA^	6.85 (±0.08)^bAB^	6.60 (±0.01)^cB^	6.10 (±0.14)^bC^	6.70 (±0.02)^aA^	6.35 (±0.05)^aB^	6.05 (±0.07)^aB^	5.70 (±0.00)^aC^	5.40 (±0.09)^bC^
CHI 4	7.10 (±0.14)^aA^	7.05 (±0.06)^abA^	6.90 (±0.02)^bA^	6.85 (±0.07)^aA^	6.05 (±0.01)^bA^	6.00 (±0.03)^bA^	5.96 (±0.03)^aA^	5.90 (±0.04)^aA^	5.85 (±0.07)^aA^
OVEO 1.25	7.08 (±0.14)^aA^	7.00 (±0.07)^abA^	6.95 (±0.07)^abA^	6.90 (±0.01)^aA^	6.00 (±0.01)^bA^	5.97 (±0.07)^bA^	5.95 (±0.02)^aA^	5.93 (±0.07)^aA^	5.75 (±0.06)^abA^
CHI 4 + OVEO 1.25	7.20 (±0.00)^aA^	7.15 (±0.05)^aA^	7.08 (±0.04)^aA^	7.05 (±0.07)^aA^	6.05 (±0.07)^bA^	5.98 (±0.04)^bA^	5.92 (±0.03)^bA^	5.90 (±0.02)^aA^	5.85 (±0.04)^aA^
Lycopene (μg/g)^*^	1–12 days				1–24 days				
	Control	CHI 4	OVEO 1.25	CHI 4 + OV 1.25	Control	CHI 4	OVEO 1.25	CHI 4 + OV 1.25	
	−8.80 (±0.02)^a^	−6.25 (±0.01)^b^	−6.24 (±0.03)^b^	−4.21 (±0.02)^c^	−7.77 (±0.04)^a^	−5.10 (±0.01)^c^	−5.64 (±0.02)^b^	−3.49 (±0.02)^d^	

Control: 0 μL/mL of chitosan and essential oil; CHI 4 + OVEO 1.25: CHI 4 mg/mL + OVEO 1.25 μL/mL. A–E: For each trial, different superscript letters in the same row denote differences (p ≤ 0.05) among the mean values (for the same treatment at different storage periods) according to Tukey's test. a–d: For each trial, different superscript letters in the same column denote differences (p ≤ 0.05) among the mean values (for the different treatments at a same storage period) according to Tukey's test.

* Mean ± standard deviations of (+) increase or decrease (−) amounts during the storage period.

The firmness decreased (*p* ≤ 0.05) with increasing storage time in coated and uncoated cherry tomato fruit. The firmness of fruit coated with CHI-OVEO was higher (*p* ≤ 0.05) in all assessed storage time points when compared with that of uncoated fruit or fruit coated with only CHI or OVEO (Table 2).

The TA and TSS decreased in uncoated fruit (*p* ≤ 0.05) stored at both room and cold temperatures, whereas no changes (*p* > 0.05) were observed in TA or TSS in fruit coated with CHI-OVEO (Table 2).

The content of lycopene decreased (*p* ≤ 0.05) in uncoated and coated fruit during storage at room or cold temperature. However, cherry tomato fruit coated with CHI-OVEO showed a minor (*p* ≤ 0.05) decrease of lycopene when compared with the other fruit groups (Table 2).

The uncoated and coated cherry tomato fruit were predominantly red throughout storage at room and cold temperatures (Supplementary Table 3). The a^*^ values decreased (*p* ≤ 0.05) over time in uncoated fruit stored at both temperatures, whereas they did not change in fruit coated with CHI-OVEO. The b^*^ values decreased (*p* ≤ 0.05) in uncoated and coated fruit during storage at room and cold temperature. The lightness values (L^*^ value) were higher (*p* ≤ 0.05) in fruit coated with CHI-OVEO or those coated only with CHI at all-time intervals assessed in both room and cold temperature storage (Supplementary Table 3).

### Effects on sugars and organic acids in cherry tomato fruit

**Figure 1:**
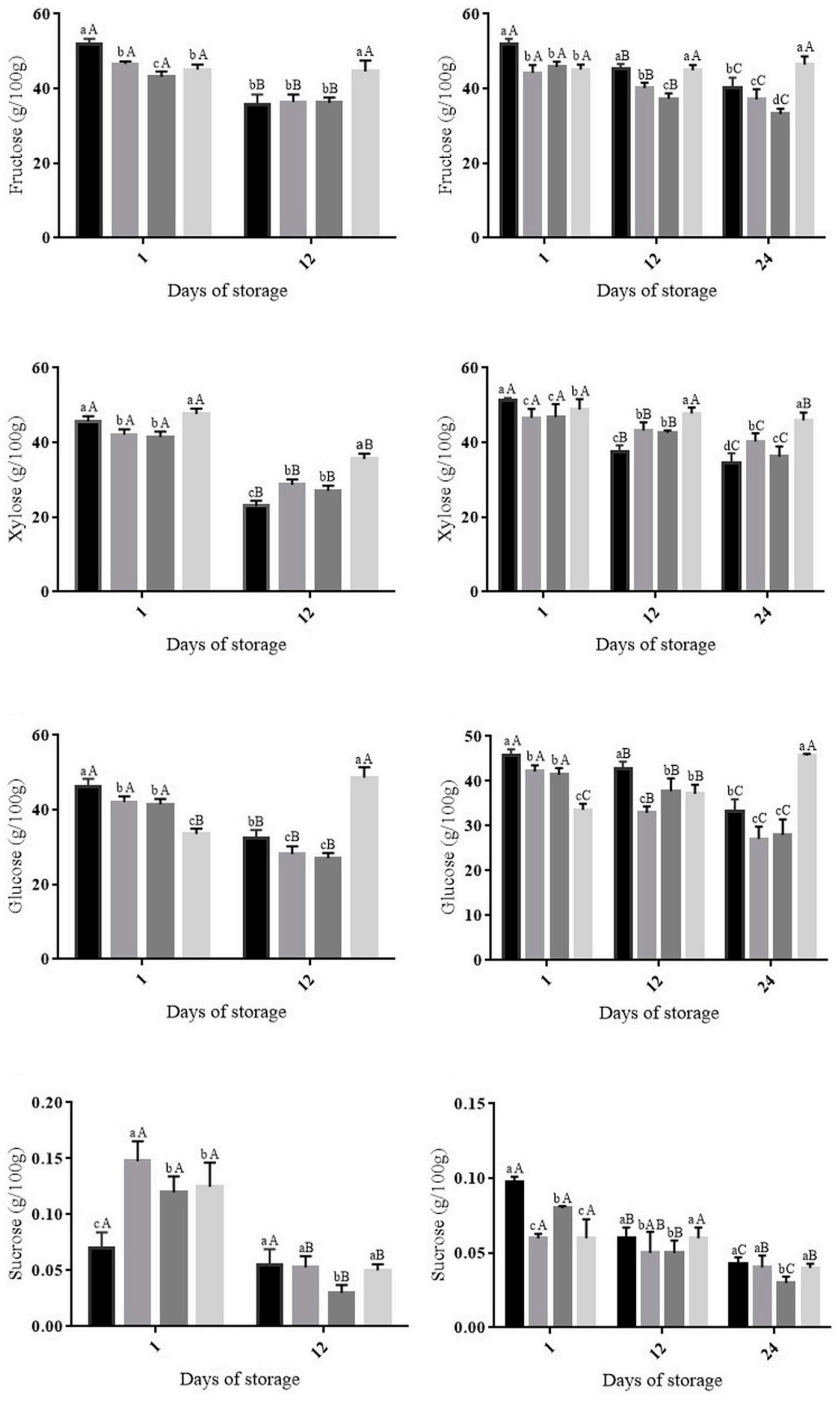
**Changes in sugar concentrations in cherry tomato fruit uncoated and coated with chitosan (CHI) and/or *O. vulgare* L. essential oil (OVEO), followed by storage at room temperature (25°C, 12 days) or cold temperature (12°C, 24 days)**. (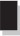) 0 μL/mL of chitosan and essential oil (control); (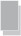) CHI 4: CHI 4 mg/mL; (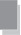) OVEO 1.25: OVEO 1.25 μL/mL; (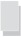) CHI 4 + OVEO 1.25: CHI 4 mg/mL + OVEO 1.25 μL/mL. A–C For each trial, different superscript letters denote differences (*p* ≤ 0.05) among the mean values (for the same treatment at different storage periods) according to Tukey's test or Student's *t-*test t. a–d: For each trial, different superscript letters denote differences (*p* ≤ 0.05) among the mean values (for the different treatments at a same storage period) according to Tukey's test.

The sugar present in the highest (*p* ≤ 0.05) amounts in uncoated or coated cherry tomato fruit was xylose (Figure 1). This sugar decreased in all fruit groups during storage at room or cold temperature; however, the greater (*p* ≤ 0.05) decrease was observed in uncoated fruit compared with fruit coated with CHI-OVEO.

Cherry tomato fruit coated with CHI-OVEO exhibited an increase in the amount of glucose (*p* ≤ 0.05) during storage at room and cold temperatures, whereas the amount of this sugar in other fruit group decreased (*p* ≤ 0.05) (Figure 1). No changes in the amount of fructose (*p* > 0.05) were observed in fruit coated with CHI-OVEO stored at room or cold temperature. Uncoated fruit or fruit coated with only CHI or OVEO stored at both temperatures showed decreases (*p* ≤ 0.05) in fructose contents (Figure 1). Sucrose amounts decreased (*p* ≤ 0.05) in all fruit groups during storage at both room and cold temperatures (Figure 1).

**Figure 2:**
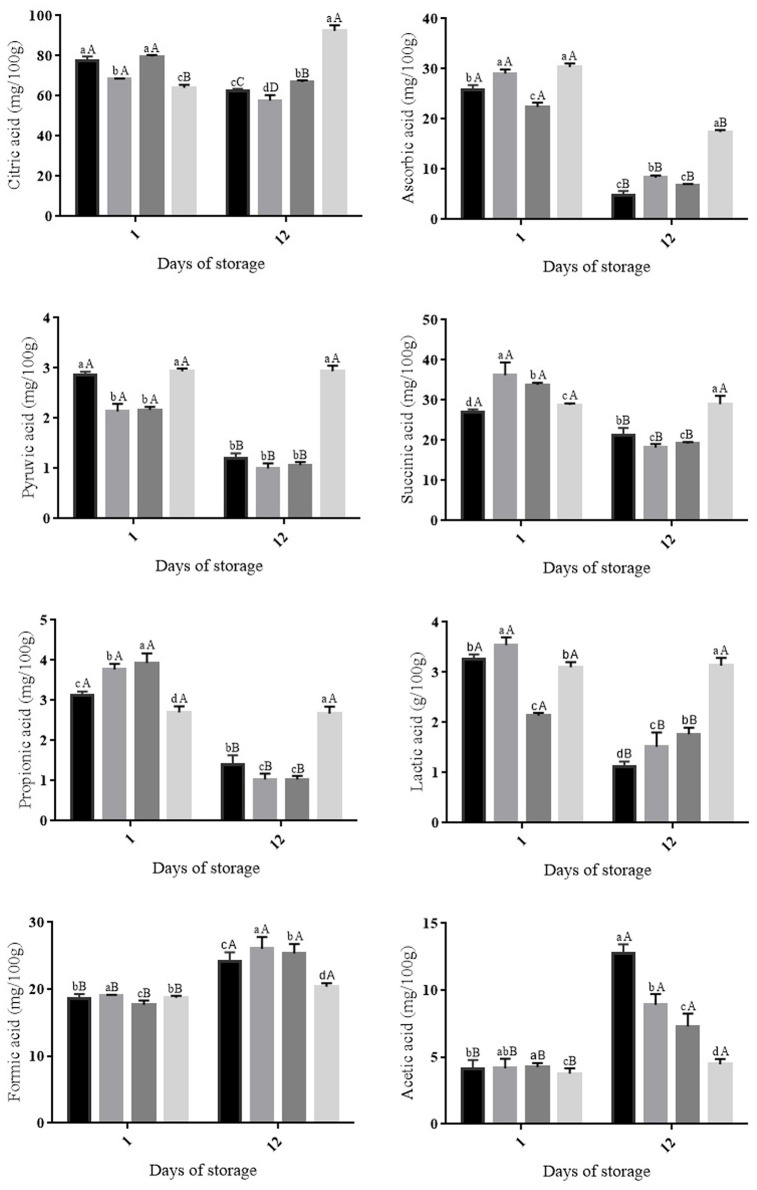
**Changes in organic acid concentrations in cherry tomato fruit uncoated and coated with chitosan (CHI) and/or *O. vulgare* L. essential oil (OVEO), followed by storage at room temperature (25°C, 12 days)**. (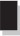) 0 μL/mL of chitosan and essential oil (control); (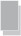) CHI 4: CHI 4 mg/mL; (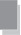) OVEO 1.25: OVEO 1.25 μL/mL; (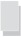) CHI 4 + OVEO 1.25: CHI 4 mg/mL + OVEO 1.25 μL/mL. A–D: For each trial, different superscript letters denote differences (*p* ≤ 0.05) among the mean values (for the same treatment at different storage periods) according to Tukey's test or Student's *t-*test. a–d: For each trial, different superscript letters denote differences (*p* ≤ 0.05) among the mean values (for the different treatments at a same storage period) according to Tukey's test.

**Figure 3:**
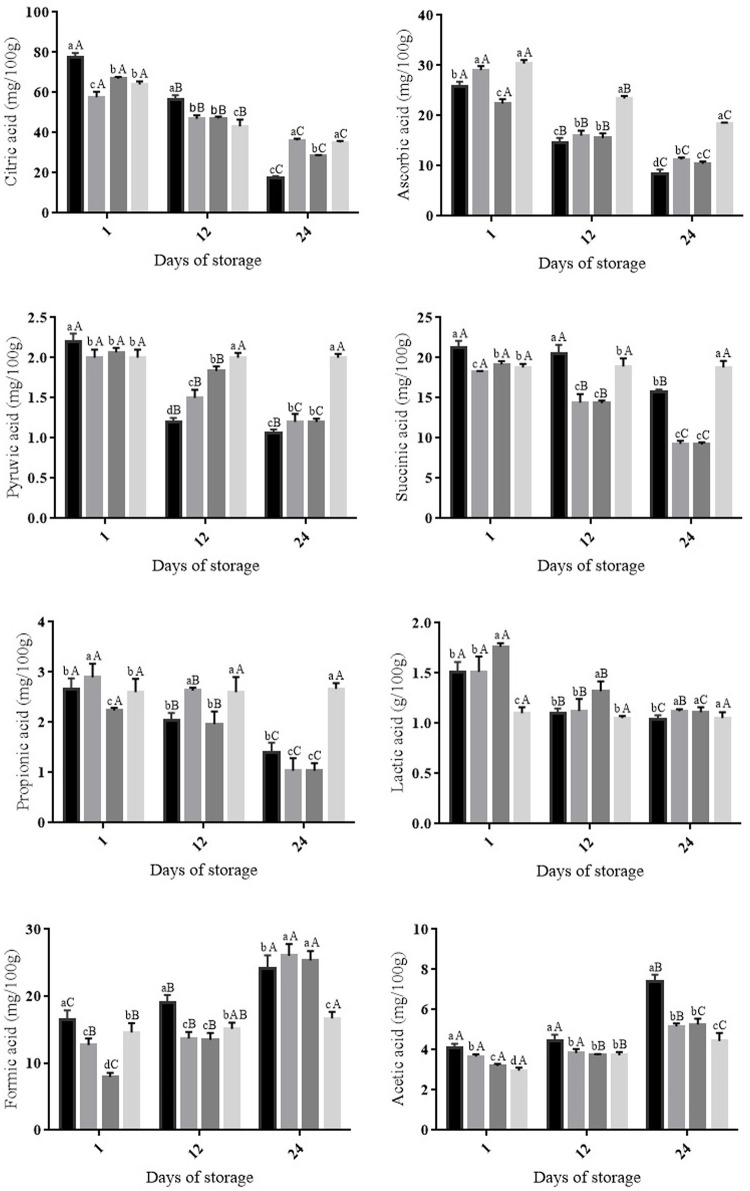
**Changes in organic acids concentrations in cherry tomato fruit uncoated and coated with chitosan (CHI) and/or *O. vulgare* L. essential oil (OVEO), followed by storage at cold temperature (12°C, 24 days)**. (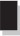) 0 μL/mL of chitosan and essential oil (control); (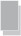) CHI 4: CHI 4 mg/mL; (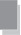) OVEO 1.25: OVEO 1.25 μL/mL; (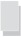) CHI 4 + OVEO 1.25: CHI 4 mg/mL + OVEO 1.25 μL/mL. A-C For each trial, different superscript letters denote differences (*p* ≤ 0.05) among the mean values (for the same treatment at different storage periods) according to Tukey's test or Student's *t*-test. a–d: For each trial, different superscript letters denote differences (*p* ≤ 0.05) among the mean values (for the different treatments at a same storage period) according to Tukey's test.

Ascorbic and citric acids were the organic acids detected in highest (*p* ≤ 0.05) amounts in all fruit groups stored at both room and cold temperatures (Figures 2, 3). The decrease (*p* ≤ 0.05) in ascorbic acid was greater in uncoated fruit or fruit coated with only OVEO or CHI than that in fruit coated with CHI-OVEO during storage at both room and cold temperature (Figures 2, 3). The amounts of citric acid decreased (*p* ≤ 0.05) in all fruit groups during cold storage, whereas it increased (*p* ≤ 0.05) in fruit coated with CHI-OVEO during storage at room temperature (Figures 2, 3).

During storage at both room temperature or cold temperatures, pyruvic, succinic, propionic and lactic acids amounts decreased (*p* ≤ 0.05) in uncoated cherry tomato fruit and in fruit coated with only CHI or OVEO. No changes in the amounts of these organic acids were observed in fruit coated with CHI-OVEO stored at room or cold temperatures (Figures 2, 3). The increases observed in the amounts of formic and acetic acids were higher (*p* ≤ 0.05) in uncoated fruit than those detected in fruit coated with CHI-OVEO during storage at room or cold temperature (Figures 2, 3).

### Effects on phenolic compounds in cherry tomato fruit

During storage at room and cold temperatures, uncoated and coated fruit exhibited decreases in the amount of 2.5-dihydroxybenzoic acid. A minor decrease (*p* ≤ 0.05) in the amount of this phenolic acid was observed in fruit coated with CHI-OVEO (Table 3). The amount of 4-dihydroxybenzoic, salicylic, syringic and caffeic acids increased (*p* ≤ 0.05) in all fruit groups during storage at both temperatures (Table 3). However, cherry tomato fruit coated with CHI-OVEO always exhibited the most significant increase (*p* ≤ 0.05) in the levels of these compounds (Table 3). A decrease (*p* ≤ 0.05) of sinapinic acid and the increase (*p* ≤ 0.05) of *p*-coumaric and *trans*-cinnamic acids amounts were similar (*p* > 0.05) among the fruit groups (Table 3). Vanillic acid amounts increased (*p* ≤ 0.05) only in fruit coated with CHI-OVEO during storage at room or cold temperature (Table 3). Ellagic acid amounts decreased (*p* ≤ 0.05) in uncoated fruit and fruit coated with only CHI or OVEO stored at both temperatures. No changes (*p* > 0.05) in the amount of this acid were observed in fruit coated with CHI-OVEO (Table 3).

**Table 3:** **Changes (increase or decrease) in phenolic compounds in cherry tomato fruit uncoated and coated with chitosan (CHI) and/or *O. vulgare* L. essential oil (OVEO), followed by storage at room temperature (25°C, 12 days) or cold temperature (25°C, 24 days)**.

Phenolic (mg/100 g)^*^	**Room temperature (25°C)**				**Cold temperature (12°C)**			
	**Control CHI 4**		**OVEO 1.25 CHI 4** + **OVEO 1.25**		**Control CHI 4**		**OVEO 1.25 CHI 4** + **OVEO 1.25**	
	**1–12 days**	**1–12 days**	**1–12 days**	**1–12 days**	**1–24 days**	**1–24 days**	**1–24 days**	**1–24 days**
4-hydroxybenzoic acid	+3.00 (±0.05)^b^	+1.00 (±0.02)^d^	+2.00 (±0.05)^c^	+5.00 (±0.02)^a^	+3.00 (±0.01)^b^	Nd	+3.00 (±0.02)^b^	+5.00 (±0.01)^a^
Salicylic acid	+11.00 (±0.01)^d^	+12.00 (±0.03)^c^	+14.00 (±0.01)^b^	+16.00 (±0.01)^a^	+13.00 (±0.03)^d^	+15.00 (±0.04)^c^	+16.00 (±0.02)^b^	+19.00 (±0.01)^a^
Sinapinic acid	−4.00 (±0.01)^a^	−3.50 (±0.02)^b^	−2.00 (±0.02)^c^	−4.00 (±0.02)^a^	−4.00 (±0.03)^c^	−6.00 (±0.01)^d^	−1.00 (±0.05)^a^	−2.00 (±0.05)^b^
Syringic acid	+4.00 (±0.02)^c^	+3.00 (±0.02)^d^	+7.00 (±0.02)^b^	+13.00 (±0.03)^a^	+2.00 (±0.05)^c^	nd	+3.00 (±0.01)^b^	+7.00 (±0.04)^a^
2.5-dihydroxybenzoic acid	−39.00 (±0.03)^d^	−22.00 (±0.04)^c^	−21.00 (±0.02)^b^	−16.00 (±0.01)^a^	−76.00 (±0.05)^d^	−35.00 (±0.01)^b^	−31.00 (±0.03)^b^	−26.00 (±0.02)^a^
Vanillic acid	nd	nd	nd	+1.00 (±0.05)^a^	nd	nd	nd	+1.00 (±0.01)^a^
Feluric acid	−3.00 (±0.05)^b^	nd	−2.00 (±0.03)^a^	−3.00 (±0.02)^b^	−3.00 (±0.01)^b^	nd	−2.00 (±0.02)^a^	−3.00 (±0.01)^b^
Ellagic acid	−1.00 (±0.02)^b^	−1.00 (±0.02)^b^	−1.00 (±0.05)^b^	nd	−1.00 (±0.02)^b^	−1.00 (±0.02)^b^	−1.00 (±0.01)^b^	nd
ρ-coumaric acid	+1.50 (±0.02)^a^	+1.40 (±0.01)^a^	+1.30 (±0.03)^a^	+1.20 (±0.01)^a^	+1.00 (±0.01)^b^	+1.00 (±0.01)^b^	+1.00 (±0.01)^b^	+1.50 (±0.01)^a^
Caffeic acid	+35.00 (±0.01)^c^	+33.00 (±0.02)^d^	+36.00 (±0.02)^b^	+37.00 (±0.04)^a^	+34.00 (±0.03)^d^	+37.00 (±0.02)^b^	+36.00 (±0.02)^c^	+41.00 (±0.05)^a^
trans-cinnamic acid	+1.00 (±0.03)^a^	+1.00 (±0.01)^a^	+1.00 (±0.01)^a^	+1.00 (±0.02)^a^	+1.00 (±0.01)^a^	+1.00 (±0.02)^a^	+1.00 (±0.03)^a^	+1.00 (±0.01)^a^
Rutin	−1.00 (±0.01)^a^	−2.00 (±0.01)^b^	−1.00 (±0.01)^a^	nd	+12.00 (±0.02)^c^	+7.00 (±0.05)^d^	+15.00 (±0.03)^b^	+17.00 (±0.04)^a^
Myricetin	+8.00 (±0.03)^b^	+4.00 (±0.01)^d^	+5.00 (±0.01)^c^	+10.00 (±0.03)^a^	+7.00 (±0.01)^c^	+5.00 (±0.05)^d^	+9.00 (±0.01)^b^	+15.00 (±0.02)^a^
Catechin	+7.00 (±0.01)^b^	+4.00 (±0.02)^d^	+5.00 (±0.01)^c^	+14.00 (±0.02)^a^	+3.00 (±0.02)^c^	+3.00 (±0.02)^c^	+6.00 (±0.03)^b^	+11.00 (±0.03)^a^
Naringenin	+2.00 (±0.02)^b^	+2.80 (±0.03)^ab^	+3.00 (±0.02)^a^	+3.00 (±0.01)^a^	+2.00 (±0.01)^b^	+2.00 (±0.01)^b^	+2.00 (±0.01)^b^	+3.00 (±0.02)^a^

* Mean ± standard deviations of (+) increase or decrease (−) of phenolic compounds observed during the storage period in mg/g of cherry tomato fruit; Control: 0 μL/mL of chitosan and essential oil CHI 4 + OVEO 1.25: CHI 4 mg/mL + OVEO 1.25 μL/mL. a–d: For each trial, different superscript letters in the same row denote differences (p ≤ 0.05) among the mean values (for the different treatments at a same storage period) according to Tukey's test; nd, not detected.

Regarding flavonoids, rutin amounts decreased (*p* ≤ 0.05) in uncoated fruit or fruit coated with only CHI or OVEO stored at room or cold temperatures; however, they did not change (*p* > 0.05) in fruit coated with CHI-OVEO (Table 3). Catechin and myricetin amounts increased in all fruit groups during storage at both temperatures; however, the greatest increases (*p* ≤ 0.05) occurred in fruit coated with CHI-OVEO (Table 3).

### Effects on sensory aspects of cherry tomato fruit

The uncoated and coated cherry tomato fruit received similar scores (*p* > 0.05) for flavor, texture, aftertaste and overall evaluation at the different storage times assessed (Supplementary Table 4). The scores fell between “liked slightly” or “liked moderately” for all sensory parameters tested. Fruit coated with CHI-OVEO received higher scores (*p* ≤ 0.05) for appearance, taste and color (corresponding to “liked very much”) in tests performed after 6 and 12 days of storage. When the panelists were asked to indicate the intent to purchase the cherry tomato fruit, the responses were generally “possibly purchase” for all fruit groups and assessed storage periods.

## Discussion

The combined incorporation of CHI and OVEO at different concentrations in growth media resulted in fungicidal effects against both *R. stolonifer* and *A. niger* and inhibited the spore germination and sporulation of these fungi over the storage time assessed. OVEO acts primarily on the fungal cell wall and, after an initial disturbance, can attack intracellular targets causing thinning and wrinkling of mycelia upon the loss of cytoplasmic material, resulting in mycelium death and lack of sporulation (Vesentini et al., 2007; Moreira et al., 2010). CHI can change the fungal membrane permeability through interactions with the negatively charged phospholipids of the plasma membrane. The enhanced antifungal effects observed for the combined application of CHI and OVEO likely occurred because the initial disturbance of the cell exterior is promoted by CHI, which facilitates cellular uptake of OVEO (Ojagh et al., 2010; Santos et al., 2012).

The CHI-OVEO coating delayed the occurrence of black mold and soft rot in cherry tomato fruit during storage at room and cold temperatures and reduced the incidence of rotten fruit at the end of storage. These results support the antifungal effects observed in *in vitro* assays. Interestingly, antifungal efficiency of CHI combined with EOs has not always been observed in *in vitro* studies with fruit, probably due to the high volatility of the EOs constituents and the possible interactions between the coating components and the vegetative tissue (Cháfer et al., 2012; Perdones et al., 2012; Shao et al., 2015).

Control of postharvest fungal diseases using CHI-coatings containing EOs seems to occur through a direct inhibitory effect on fungi cells and an indirect effect of inducing defense mechanisms in the fruit tissue (Aquino et al., 2015; Shao et al., 2015). It has been proposed that the efficacy of CHI-EOs in inhibiting pathogenic fungi in fruit could be partially related to their ability to induce production of defense-related enzymes, e.g., polyphenoloxydase, peroxidase, chitinase, and β-1,3-glucanase, in coated fruits (Shao et al., 2015).

Greater antifungal efficacy was observed when CHI-OVEO coating was applied in cherry tomato fruit stored at cold temperature. These results are probably associated with the slowing of physiological maturation processes in fruit stored at cold temperatures, which consequently led to a higher resistance to mold infection (Sánchez-González et al., 2011). Another important reason could be the weaker pathogenicity of the fungal strains at low temperatures because the antifungal activity of CHI and EOs on fruit increases with decreased storage temperature (Santos et al., 2012; de Oliveira et al., 2014).

Fruit coated with CHI-OVEO had higher firmness and lower weight loss during storage at room or cold temperatures. The firmness of fruit during storage is directly related to softening resulting from the cell wall modification caused by the degradation of pectin during respiration (Ali et al., 2010). Otherwise, weight loss in fruit is primarily associated with the water loss caused by transpiration and respiration (Wu et al., 2016). The observed results are probably related to the effects of CHI-OVEO coating as a semi-permeable barrier to O_2_, CO_2_, water and solutes (Aquino et al., 2015). Because of its hydrophobicity, OVEO improved the physical barrier properties of CHI-coatings (Perdones et al., 2012). Consequently, the respiration rates may be decreased in fruit coated with CHI-OVEO, as well as the water loss and softening. The antifungal effects of the CHI-OVEO coating may also contribute to the maintenance of firmness because this coating may protect the fruit against fungal cell wall-degrading enzymes used for colonization and infection (de Oliveira et al., 2014).

TA decreased in uncoated cherry tomato fruit, whereas fruit coated with CHI-OVEO did not change this parameter during storage at both studied temperatures. The TA decreases with the fruit ripening; however, a delay of this decrease is interesting because low amounts of TA induce faster senescence of fruit (Khaliq et al., 2015). TA indexes are related to amounts of organic acids present in tomato fruit, and acidity found in the early stages of maturation tends to decrease by degradation of these compounds (Wu et al., 2016). Organic acids are used as substrates for enzymatic reactions, as a major source of ATP, or as intermediate metabolites in biochemical reactions during fruit respiration (Khaliq et al., 2015; Wu et al., 2016). In the present study, organic acids directly related to respiration metabolism (e.g., citric, pyruvic, succinic and lactic acids) decreased only in uncoated fruit during the storage at both room and cold temperatures, explaining the observed TA decrease (Wu et al., 2016). Because the metabolism was slowed, the degradation of organic acids in fruit coated with CHI-OVEO was reduced and contributed to maintenance of the TA values during storage. Additionally, the increase of formic and acetic acids amounts only in uncoated fruit indicated the effectiveness of the CHI-OVEO coating to delay the senescence in cherry tomato fruit. Overall, the CHI-OVEO coating slowed the decrease of ascorbic acid levels, an important bioactive compound of cherry tomato fruit. The inhibition of gas exchange between the fruit tissues and the environment near the CHI-OVEO coating probably reduced the O_2_ penetration and, consequently, the oxidation of ascorbic acid (Shao et al., 2015).

The TSS amounts decreased in uncoated fruit and did not change in fruit coated with CHI-OVEO during storage at room or cold temperature. Sugars are the major components of the TSS in tomato fruit (Wu et al., 2016). The CHI-OVEO coating most likely prevented the reduction of TSS because it delayed the hydrolysis of carbohydrates to sugars due the slower ripening (Elsabee and Abdou, 2013). The reduction in fruit metabolism could also explain the higher amounts of glucose and fructose observed in fruit coated with CHI-OVEO because these sugars are the predominant sugars metabolized during fruit respiration (Beckles, 2012; D'Aquino et al., 2016). The higher content of glucose, fructose, and citric acid in fruit coated with CHI-OVEO could explain the higher scores attributed to this fruit group for “taste” in the sensory test. They likely exhibited a better balance between sugars and acids because the ratio of sugars to acids defines the taste of a ripe tomato fruit (Beckles, 2012).

The application of CHI-OVEO positively affect the color of the cherry tomato fruit because the coating helped maintain the red intensity (a^*^ values) during storage. The red color of the in cherry tomato fruit is one of the most important quality criteria used in market or by consumers to judge the commercial quality of the fruit (Fagundes et al., 2015b). The increased red color observed in the cherry tomato fruit coated with CHI-OVEO was most likely related to the higher quantities of lycopene present in those fruit during storage. Lycopene is the main colored carotenoid in red tomato fruit, and these fruit are the most important sources of lycopene in the human diet (Stinco et al., 2013). The efficacy of CHI-OVEO coating in slowing the degradation of lycopene is noteworthy and similar to that observed for ascorbic acid. These observations were probably related to the barrier for gas exchange and slower metabolism. Increased respiration rates result in the ability of reactive oxygen groups to oxidize the lycopene (Shi, 2008).

The cherry tomato fruit coated with CHI-OVEO or only with CHI presented increase in lightness (L^*^) compared to the uncoated fruit or fruit coated with only OVEO. Greater lightness/brightness has been described for fruit coated with polymer-based dispersions, which can be associated with characteristics brightness and high transparency of the formed coating (de Oliveira et al., 2014). This increased lightness of the CHI and CHI-OVEO coated fruit was positively perceived by panelists who assigned higher scores for appearance and color attributes to fruit from these groups.

The phenolic acids and flavonoids detected in cherry tomato fruit were similar among the uncoated or coated fruit. However, the CHI-OVEO coating delayed any decreases and even increased specific phenolic acids or flavonoids. It is known that during the tomato fruit maturation, a variety of phenolic compounds are synthetized by hydroxylation, methylation and dehydrogenation reactions (Sánchez-Rodríguez et al., 2012; Bicudo et al., 2014; Choi et al., 2014). The synthesis or degradation of phenolic compounds is clearly associated with the ripening process, and the regulatory events in climacteric fruit (e.g., cherry tomato) depend on the action of ethylene (Alexander and Grierson, 2002). Thus, the CHI-OVEO coating interfered within the metabolism of phenolic compounds in cherry tomato fruit likely due to the creation of an internal modified atmosphere leading to the biosynthesis and accumulation of these secondary metabolites (Beckles, 2012). Overall, fruit coated with CHI-OVEO exhibited higher amounts of several phenolic compounds at the end of storage at both room and cold temperatures. Thus, given the knowledge that phenolic compounds are related to protection against fungal infection in plant tissues (Pane et al., 2016), the CHI-OVEO coating could enhance the resistance to pathogenic fungi in cherry tomato fruit during storage.

## Conclusions

CHI and OVEO in combination inhibited spore germination and showed fungicidal *in vitro* effects against *R. stolonifer* and *A. niger*. The application of CHI-OVEO coating delayed the mold decay in cherry tomato fruit and enhanced important quality and sensory aspects of these fruit during storage at room or cold temperatures. The tested CHI-OVEO increased the amounts of phenolic compounds retained during the storage time assessed. CHI-OVEO coating is a feasible treatment for maintaining the quality of cherry tomato fruit during storage.

## Author contributions

The authors TB, SA, and NA, contributed to acquisition, analysis, and interpretation of data. The authors MSM, and JM contributed to interpretation of data and critically revised manuscript. The authors BM and AC contributed to acquisition and analysis of data. ES and MM contributed to conception and design, as well as the interpretation of data. Also they drafted and critically revised the manuscript.

### Conflict of interest statement

The authors declare that the research was conducted in the absence of any commercial or financial relationships that could be construed as a potential conflict of interest.
