# Supplementary material for: A Chitosan Coating Containing Essential Oil from Origanum vulgare L. to Control Postharvest Mold Infections and Keep the Quality of Cherry Tomato Fruit
Source: Front Microbiol. 2016 Nov 8;7:1724. doi: 10.3389/fmicb.2016.01724 (PMC5099165; doi:10.3389/fmicb.2016.01724)
Supplement: Supplementary file 1 [file Table1.DOCX]

**Supplementary Table 1.** GC–MS analysis of the essential oil *Origanum vulgare* L.

| Peaks | Constituents | Area (%)^*^ |
| --- | --- | --- |
| 1 | 2-hexanone | 0.04 |
| 2 | 2- hexanone, 3,3-dimethyl | 0.57 |
| 3 | ethanone 1-(3-ethyloxiranyl) | 0.42 |
| 4 | *α*-thujene | 0.36 |
| 5 | *α*- pinene | 1.69 |
| 6 | bicyclo[2.2.1]heptane, 2,2 dimethyl-3-methyl | 0.57 |
| 7 | 3-hexen-2-one | 0.29 |
| 8 | β-pinene | 0.69 |
| 9 | myrcene | 1.15 |
| 10 | pseudolimonene | 0.12 |
| 11 | α-terpinene | 0.93 |
| 12 | σ-cymol | 12.50 |
| 13 | limonene | 0.11 |
| 14 | eucalyptol | 0.30 |
| 15 | *y-*terpinene | 6.78 |
| 16 | linalool | 2.85 |
| 17 | thymolmethylether | 0.17 |
| 18 | anethole | 0.60 |
| 19 | thymol | 3.82 |
| 20 | carvacrol | 64.42 |
| 21 | caryophyllene (E) | 1.23 |
| 22 | caryophyllene oxide | 0.39 |

_*_- The quantification of the constituents was obtained after normalizing the areas of each detected constituent, expressed as a percentage area (%).

**Supplementary Table 2.** Effects of chitosan (CHI) and/or *O. vulgare* L. essential oil (OVEO) alone or in combination on *R. stolonifer* URM 3728 and *A. niger* URM 5842 spore germination and sporulation after 24 h-incubation (25 °C).

| Treatments | Inhibition of spore germination (%) | |  | N° spores/ mL (10^4^) | |
| --- | --- | --- | --- | --- | --- |
|  | *R. stolonifer* | *A. niger* |  | *R. stolonifer* | *A. niger* |
| Control | 0.00% (±0.00)^c^ | 0.00% (±0.00)^d^ |  | 18.20(±0.10)^a^ | 75.63(±0.30)^a^ |
| CHI 4 | 82.00% (±0.10)^ab^ | 26.00 (±0.10)^b^ |  | 16.60(±0.25)^b^ | 36.80(±0.40)^b^ |
| OVEO 1.25 | 61.00% (±0.10)^b^ | 11.00 (±0.10)^c^ |  | 0.00(±0.00)^c^ | 0.00(±0.00)^c^ |
| CHI 4 + OVEO 5 | 84.00% (±0.10)^a^ | 100.00% (±0.00)^a^ |  | 0.00(±0.00)^c^ | 0.00(±0.00)^c^ |
| CHI 4 + OVEO 2.5 | 83.00% (±0.30)^a^ | 90.00% (±0.10)^a^ |  | 0.00(±0.00)^c^ | 0.00(±0.00)^c^ |
| CHI 4 + OVEO 1.25 | 75.00% (±0.20)^ab^ | 90.00% (±0.10)^a^ |  | 0.00(±0.00)^c^ | 0.00(±0.00)^c^ |

The results expressed as percent inhibition rates of spore germination compared with the control treatment (0 μL/mL of chitosan and essential oil). CHI 4 + OVEO 5: CHI 4 mg/ mL+ OVEO 5 μL/ mL; CHI 4 + OVEO 2.5: CHI 4 mg/ mL + OVEO 2.5 μL/ mL; CHI 4 + OVEO 1.25: CHI 4 mg/ mL + OVEO 1.25 μL/ mL; a–d: For each trial, different superscript letters in the same column denote differences (p ≤ 0.05) among the mean values (for each fungus submitted for the different treatments) according to Tukey's test.

**Supplementary Table 3.** Mean values of the color in cherry tomato fruit uncoated and coated with chitosan (CHI) and/or *O. vulgare* L. essential oil (OVEO), followed by storage at room temperature (25 °C, 12 days) or cold temperature (12 °C, 24 days).

| Parameters | Days of storage | Treatments | | | |
| --- | --- | --- | --- | --- | --- |
|  |  | Control | CHI 4 | OVEO 1.25 | CHI 4 + OVEO 1.25 |
| Room temperature | | | | | |
| L | 0 | 35.70 (±0.18)^ab B^ | 35.21 (±0.21)^b C^ | 36.27 (±0.13)^a B^ | 35.02 (±0.08)^b C^ |
|  | 4 | 35.93 (±0.23)^ab B^ | 35.51 (±0.07)^b C^ | 36.74 (±0.12)^a B^ | 36.45 (±0.19)^ab B^ |
|  | 8 | 37.33 (±0.22)^a A^ | 37.13 (±0.20)^a B^ | 37.01 (±0.21)^a AB^ | 36.89 (±0.17)^a B^ |
|  | 12 | 38.18 (±0.12)^b A^ | 41.71 (±0.22)^a A^ | 37.94 (±0.11)^b A^ | 42.26 (±0.18)^a A^ |
| a | 0 | 17.32 (±0.16)^a A^ | 13.88 (±0.17)^bc A^ | 13.08 (±0.02)^c A^ | 14.28 (±0.08)^b A^ |
|  | 4 | 16.77 (±0.22)^a A^ | 14.16 (±0.18)^b A^ | 12.96 (±0.06)^c A^ | 14.17 (±0.16)^b A^ |
|  | 8 | 13.85 (±0.10)^a B^ | 14.37 (±0.10)^a A^ | 13.00 (±0.15)^b A^ | 14.10 (±0.18)^a A^ |
|  | 12 | 11.90 (±0.06)^c C^ | 14.37 (±0.09)^a A^ | 13.04 (±0.03)^b A^ | 14.07 (±0.13)^a A^ |
| b | 0 | 19.90 (±0.17)^a A^ | 18.07 (±0.15)^b A^ | 19.61 (±0.04)^a A^ | 18.66 (±0.08)^b A^ |
|  | 4 | 18.73 (±0.07)^a B^ | 17.88 (±0.06)^a A^ | 18.07 (±0.16)^a B^ | 17.83 (±0.05)^a B^ |
|  | 8 | 17.30 (±0.14)^b C^ | 17.62 (±0.07)^ab A^ | 18.05 (±0.12)^a B^ | 17.14 (±0.16)^b B^ |
|  | 12 | 15.77 (±0.09)^bc D^ | 15.20 (±0.16)^c B^ | 17.42 (±0.23)^a C^ | 16.00 (±0.09)^b C^ |
| C*ab | 0 | 21.11 (±0.13)^a B^ | 22.85 (±0.12)^a AB^ | 23.09 (±0.11)^a AB^ | 23.63 (±0.13)^a A^ |
|  | 4 | 23.69 (±0.06)^a AB^ | 24.10 (±0.24)^a A^ | 22.35 (±0.16)^a B^ | 22.40 (±0.17)^a AB^ |
|  | 8 | 25.73 (±0.20)^a A^ | 24.32 (±0.16)^a A^ | 26.27 (±0.21)^a A^ | 24.14 (±0.02)^a A^ |
|  | 12 | 23.45 (±0.15)^a AB^ | 20.85 (±0.18)^ab B^ | 21.71 (±0.20)^ab B^ | 20.24 (±0.14)^b B^ |
| h*ab | 0 | 55.70 (±0.01)^a A^ | 52.66 (±0.20)^a A^ | 51.51 (±0.22)^a A^ | 53.00 (±0.13)^a A^ |
|  | 4 | 53.57 (±0.11)^ab AB^ | 47.27 (±0.18)^b A^ | 54.74 (±0.19)^a A^ | 50.96 (±0.05)^ab A^ |
|  | 8 | 49.91 (±0.04)^a B^ | 47.93 (±0.24)^a A^ | 49.96(±0.06)^a A^ | 48.23 (±0.16)^a A^ |
|  | 12 | 43.53 (±0.13)^c C^ | 47.16 (±0.15)^bc A^ | 54.30 (±0.09)^a A^ | 53.16 (±0.23)^ab A^ |
| Cold temperature | |  |  |  |  |
| L | 0 | 36.08 (±0.12)^a A^ | 34.86 (±0.07)^b C^ | 35.63 (±0.13)^ab D^ | 33.94 (±0.5)^c D^ |
|  | 6 | 36.33 (±0.08)^a A^ | 35.08 (±0.13)^b C^ | 36.22 (±0.18)^a CD^ | 35.51 (±0.12)^b C^ |
|  | 12 | 36.76 (±0.13)^b A^ | 35.98 (±0.22)^b B^ | 36.91 (±0.09)^b BC^ | 38.05 (±0.18)^a B^ |
|  | 18 | 36.86 (±0.18)^bc A^ | 36.13 (±0.21)^c B^ | 37.16 (±0.03)^b AB^ | 38.59 (±0.15)^a B^ |
|  | 24 | 36.94 (±0.09)^c A^ | 38.15 (±0.19)^b A^ | 37.95 (±0.16)^b A^ | 40.24 (±0.04)^a A^ |
| a | 0 | 15.72 (±0.15)^a A^ | 13.10 (±0.08)^b A^ | 12.20 (±0.07)^c A^ | 12.98 (±0.16)^b A^ |
|  | 6 | 15.13 (±0.08)^a AB^ | 13.30 (±0.10)^b A^ | 12.21 (±0.14)^c A^ | 13.00 (±0.14)^b A^ |
|  | 12 | 14.73 (±0.18)^a B^ | 13.07 (±0.13)^b A^ | 12.21 (±0.16)^c A^ | 13.04 (±0.09)^b A^ |
|  | 18 | 13.02 (±0.19)^ab C^ | 13.09 (±0.09)^a A^ | 12.25 (±0.11)^b A^ | 13.07 (±0.10)^a A^ |
|  | 24 | 12.26 (±0.20)^b C^ | 13.15 (±0.15)^a A^ | 12.26 (±0.07)^b A^ | 13.84 (±0.14)^ab A^ |
| b | 0 | 22.51 (±0.05)^a A^ | 20.95 (±0.17)^b A^ | 21.19 (±0.21)^b A^ | 19.75 (±0.04)^c A^ |
|  | 6 | 22.05 (±0.16)^a A^ | 20.17 (±0.06)^c B^ | 20.80 (±0.13)^b AB^ | 18.95 (±0.15)^d B^ |
|  | 12 | 20.18 (±0.04)^a B^ | 19.17 (±0.07)^b C^ | 20.18 (±0.08)^a B^ | 18.67 (±0.16)^b B^ |
|  | 18 | 17.44 (±0.12)^c C^ | 18.72 (±0.12)^b C^ | 20.15 (±0.14)^a B^ | 18.55 (±0.13)^b BC^ |
|  | 24 | 17.15 (±0.11)^b C^ | 17.12 (±0.13)^b D^ | 18.05 (±0.19)^a C^ | 18.17 (±0.09)^a C^ |
| C*ab | 0 | 21.15 (±0.15)^a C^ | 21.56 (±0.21)^a B^ | 21.01 (±0.13)^a B^ | 23.38 (±0.08)^a A^ |
|  | 6 | 21.89 (±0.11)^a BC^ | 22.16 (±0.16)^a B^ | 23.38 (±0.07)^a AB^ | 24.86 (±0.04)^a A^ |
|  | 12 | 25.07 (±0.18)^a AB^ | 24.27 (±0.12)^a AB^ | 24.64 (±0.09)^a AB^ | 22.83 (±0.19)^a A^ |
|  | 18 | 27.49 (±0.02)^a A^ | 24.40 (±0.05)^a AB^ | 26.44 (±0.14)^a A^ | 23.66 (±0.15)^a A^ |
|  | 24 | 26.89 (±0.12)^a A^ | 26.53 (±0.18)^a A^ | 25.58 (±0.13)^ab A^ | 23.59 (±0.22)^b A^ |
| h*ab | 0 | 54.65 (±0.06)^ab A^ | 52.80 (±0.16)^b A^ | 60.91 (±0.15)^a A^ | 53.13 (±0.16)^b A^ |
|  | 6 | 53.40 (±0.15)^a A^ | 58.92 (±0.10)^a A^ | 58.53 (±0.16)^a AB^ | 52.74 (±0.12)^a A^ |
|  | 12 | 54.11 (±0.20)^a A^ | 57.06 (±0.09)^a A^ | 59.93 (±0.12)^a AB^ | 54.85 (±0.15)^a A^ |
|  | 18 | 55.43 (±0.05)^a A^ | 52.58 (±0.13)^a A^ | 52.02 (±0.08)^a B^ | 50.45 (±0.08)^a A^ |
|  | 24 | 55.96 (±0.21)^a A^ | 52.30 (±0.22)^a A^ | 53.07 (±0.13)^a AB^ | 54.12 (±0.15)^a A^ |

Control: 0 μL/mL of chitosan and essential oil; CHI 4 + OVEO 1.25: CHI 4 mg/mL + OVEO 1.25 μL/mL. A-D: For each trial, different superscript letters in the same column denote differences (p ≤ 0.05) among the mean values (for the same treatment at different storage periods) according to Tukey's test. a-c: For each trial, different superscript letters in the same row denote differences (p ≤ 0.05) among the mean values (for the different treatments at a same storage period) according to Tukey's test.

**Supplementary Table 4.** Mean values of the sensory attributes in cherry tomato fruit uncoated and coated with chitosan (CHI) and/or *O. vulgare* L. essential oil (OVEO), followed by storage at room temperature (25 °C, 12 days) or cold temperature (12 °C, 24 days).

| Attributes | Storage time (days) | Control | CHI 4 | OVEO 1.25 | CHI 4 + OVEO 1.25 |
| --- | --- | --- | --- | --- | --- |
| Appearance | 0 | 7.93 (±0.13)^a B^ | 7.87 (±0.02)^a C^ | 8.10 (±0.10)^aA^ | 7.73 (±0.13)^a B^ |
|  | 6 | 8.03 (±0.15)^bB^ | 8.23 (±0.07)^b A^ | 8.17 (±0.13)^b A^ | 8.80 (±0.14)^aA^ |
|  | 12 | 8.20 (±0.06)^b A^ | 8.00 (±0.11)^bB^ | 8.00 (±0.04)^b A^ | 8.60 (±0.01)^aA^ |
| Color | 0 | 7.70 (±0.05)^a B^ | 7.70 (±0.22)^a B^ | 7.77 (±0.16)^a B^ | 7.50 (±0.14)^a B^ |
|  | 6 | 8.03 (±0.15)^b AB^ | 8.07 (±0.17)^b A^ | 8.07 (±0.13)^b A^ | 8.95 (±0.17)^aA^ |
|  | 12 | 8.30 (±0.09)^b A^ | 7.90 (±0.26)^b AB^ | 8.10 (±0.01)^b A^ | 8.89 (±0.07)^aA^ |
| Flavor | 0 | 7.20 (±0.22)^a B^ | 6.97 (±0.11)^a B^ | 7.20 (±0.16)^aA^ | 7.30 (±0.19)^a B^ |
|  | 6 | 7.77 (±0.07)^aA^ | 7.70 (±0.19)^aA^ | 7.70 (±0.19)^aA^ | 7.87 (±0.17)^a AB^ |
|  | 12 | 8.20 (±0.19)^aA^ | 7.80 (±0.06)^aA^ | 7.70 (±0.09)^aA^ | 8.00 (±0.21)^a B^ |
| Taste | 0 | 7.43 (±0.24)^a B^ | 7.43 (±0.16)^aA^ | 7.67 (±0.18)^aA^ | 7.37 (±0.16)^a B^ |
|  | 6 | 7.50 (±0.14)^bB^ | 7.73 (±0.21)^b A^ | 7.60 (±0.19)^b A^ | 8.70 (±0.17)^aA^ |
|  | 12 | 7.90 (±0.16)^b A^ | 7.67 (±0.14)^b A^ | 7.40 (±0.07)^b A^ | 8.37 (±0.14)^aA^ |
| Aftertaste | 0 | 7.40 (±0.23)^aA^ | 7.13 (±0.17)^aA^ | 7.47 (±0.14)^aA^ | 7.00 (±0.13)^a B^ |
|  | 6 | 7.73 (±0.08)^aA^ | 7.50 (±0.12)^aA^ | 7.37 (±0.11)^aA^ | 7.60 (±0.17)^aA^ |
|  | 12 | 7.80 (±0.19)^aA^ | 7.40 (±0.23)^ab A^ | 7.20 (±0.01)^b A^ | 7.80 (±0.06)^aA^ |
| Firmness | 0 | 7.50 (±0.20)^a B^ | 7.37 (±0.06)^aA^ | 7.83 (±0.13)^aA^ | 7.50 (±0.16)^aA^ |
|  | 6 | 7.53 (±0.17)^a B^ | 7.77 (±0.20)^aA^ | 7.73 (±0.08)^aA^ | 7.63 (±0.09)^aA^ |
|  | 12 | 8.20 (±0.19)^aA^ | 7.70 (±0.22)^aA^ | 7.70 (±0.09)^aA^ | 8.00 (±0.09)^aA^ |
| Overall evaluation | 0 | 7.57 (±0.07)^aA^ | 7.53 (±0.12)^aA^ | 7.43 (±0.17)^aA^ | 7.03 (±0.16)^a B^ |
|  | 6 | 7.63 (±0.19)^aA^ | 7.67 (±0.16)^aA^ | 7.63 (±0.11)^aA^ | 7.70 (±0.09)^aA^ |
|  | 12 | 7.80(±0.16)^aA^ | 7.30(±0.22)^aA^ | 7.40(±0.23)^aA^ | 7.73(±0.21)^aA^ |
|  |  |  |  |  |  |

Control: 0 μL/mL of chitosan and essential oil; CHI 4 + OVEO 1.25: CHI 4 mg/mL + OVEO 1.25 μL/mL. A-C: For each trial, different superscript letters in the same column denote differences (p ≤ 0.05) among the mean values (for the same treatment at different storage periods) according to Tukey's test. a-b: For each trial, different superscript letters in the same row denote differences (p ≤ 0.05) among the mean values (for the different treatments at a same storage period) according to Tukey's test.
